# Neurology Case Reporting: a call for all

**DOI:** 10.1186/1752-1947-5-113

**Published:** 2011-03-23

**Authors:** Richard A Rison

**Affiliations:** 1 Clinical Assistant Professor of Neurology University of Southern California Keck School of Medicine Los Angeles County Medical Center Medical Director Presbyterian Intercommunity Hospital Stroke Center Presbyterian Intercommunity Hospital 12401 Washington Blvd. Whittier, California 90602, USA

## Abstract

From antiquity to present day, the act of recording and publishing our observations with patients remains essential to the art of medicine and the care of patients. Neurology is rich with case reports over the centuries. They contribute to our understanding and knowledge of disease entities, and are a cornerstone of our professional development as physicians and the care of our patients. This editorial seeks to enthuse and invigorate house staff and practicing physicians everywhere to continue the long and time-honored tradition of neurology case reporting.

## 

Neurology Case Reporting: A call for all

"If thou examinest a man having a wound in his temple, penetrating to the bone, (and) perforating his temporal bone;...if thou ask of him concerning his malady and he speak not to thee; while copious tears fall from both his eyes, so that he thrusts his hand often to his face so that he may wipe both his eyes with the back of his hand..."

Edwin Smith surgical papyrus,

Case 20, c2800 BC [1]

From the earliest description of aphasia by an Egyptian surgeon over 4000 years ago [1], to Journal of Medical Case Reports' (JMCR) latest neurological publication on videofluoroscopy in a patient with dysphagia as the initial presentation of myasthenia gravis currently published around the time of this writing [2], case reports have been a time-honored tradition in the neurological sciences. From daily interactions with experienced colleagues and caring for our individual patients, physicians continue to build a clinical knowledge base. This acumen must be passed on throughout the generations to better patient care. There is no better enduring method for this than the act of publication.

From antiquity to present day, the act of recording and publishing our observations with patients remains essential to the art of medicine and the care of patients. Osler stated "Always note and record the unusual... Publish it. Place it on permanent record as a short, concise note. Such communications are always of value." [3]. If we as clinicians are to know where we are headed and improve the care of the people charged to us, then we must know the past and where our predecessors have been. Case reports and history are essential dimensions. The past allows us to enlighten our perspective of the present and the future. As Osler so eloquently said:

"The past is always with us, never to be escaped; it alone is enduring; but amidst the changes and chances which succeed one another so rapidly in life, we are apt to live too much for the present and too much in the future." [4]

Neurology is replete with rich case reports over the centuries. They contribute to our understanding and knowledge of disease entities. Some of the greatest case reports in history have been neurological. Take for example our understanding of vascular neurology. Hippocrates (circa 400 BC) was a keen observer who urged careful observation and recording of phenomenology, and was among the first to write about cerebrovascular disease. Through astute observations and recording he noted "Persons are most subject to apoplexy between the ages of forty and sixty" [5] "when persons in good health are suddenly seized with pains in the head and straightaway are laid down speechless and breathe with stertor, they die in seven days when fever comes on." [6]. Such was the first known description of subarachnoid hemorrhage and remains apt to this day. Consider another recorded observation from Hippocrates, this time the first known description of a migrainous visual aura:

"Phaenix's complaint was of such a nature, that flashes like lightning seemed to dart from his eye, and generally his right eye. Not long after, a violent pain seized his right temple, and then his whole head and neck. The back part of his head at the vertebrae swelled; and the tendons were upon the stretch and hard. Now if he attempted to move his head, or to open his teeth, a pain seized him from the violence of the stretch. Vomitings, whenever they happened, removed the pains now mentioned, or made them easier..." [7]

A good story stays with you for life. Case reports are an essential complement to textbook reading and are an ideal venue for student and resident education. A whole new light is shone when one has to sit down, collect one's thoughts, and then write. Case reporting is indispensable for house staff, who should be encouraged to produce scholarly work during their training [8] given the high volume of patients they encounter, many of whom are bound to have reportable findings. The completion of a case report is often seen by house staff as less time-consuming than other scholarly endeavors [9,10]. One may refer to the succinct article by Wright and Kouroukis explaining how to approach a reportable case [11].

Whereas the love for the neurologic case report begins in residency, the art and desire to report and publish continues throughout the rest of the neurologist's career. Examining and treating patients, summarizing details and writing is important for professional growth and development. In my daily private practice I keep a list of interesting and reportable cases that I have seen in the office and the hospital. In my exam room, I keep a JMCR consent form readily available. When I see an appropriate patient, I often discuss with him/her the reportable nature of their neurologic condition. I have found more often than not that it is the patient who encourages me on visit after visit to write a case report and contribute to the literature. I recall one very pleasant woman who suffered the uncommon complication of a branch facial nerve palsy and subsequent brow droop following a temporal artery biopsy [12]. She readily consented to publication, and would persistently ask me during each follow-up visit when I was going to "write me up." I gave myself the deadline of a draft before her next visit so I could show it to her! Such patient-doctor interactions add to the joy of a daily neurology practice and keep us on our toes.

One can argue that it has never been easier to publish a case report. The growth of electronic medical journals on the Internet, which are less constrained with respect to space, provide additional opportunities for the publication of case reports [11]. Journal of Medical Case Reports is a peer-reviewed journal committed only to case reports. It is open access and remains at the forefront of clinical knowledge determination via case reporting by publishing high quality manuscripts [13]. It is an ideal venue for both neurology residents and practicing attendings to publish and carry on the tradition of neurologic case reporting. Through promoting the role of case reports in neurology, a large database of online case reports will add to the evidence-based medical literature [14], be of help to our present and future patients, and allow us to stand on the shoulders of the giants before us.

And lest one worry about an "impact factor" [13], the words of Milos Jenicek should be always remembered: "Case reports and case series may be the 'lowest' or 'weakest' level of evidence, but they often remain the 'first line of evidence'. This is where everything begins." [15].

## Competing interests

RAR serves as a Deputy Editor for *Journal of Medical Case Reports*, *Case Reports in Neurology*, *Grand Rounds*, and previously *Cases Journal*.
